# Treatment of Pheochromocytoma Cells with Recurrent Cycles of Hypoxia: A New Pseudohypoxic In Vitro Model

**DOI:** 10.3390/cells11030560

**Published:** 2022-02-05

**Authors:** Jana Helm, Stephan Drukewitz, Isabel Poser, Susan Richter, Markus Friedemann, Doreen William, Hermine Mohr, Svenja Nölting, Mercedes Robledo, Stefan R. Bornstein, Graeme Eisenhofer, Nicole Bechmann

**Affiliations:** 1 Department of Medicine III, University Hospital Carl Gustav Carus, Technische Universität Dresden, Fetscherstrasse 74, 01307 Dresden, Germany; jana.helm@uniklinikum-dresden.de (J.H.); stefan.bornstein@uniklinikum-dresden.de (S.R.B.); graeme.eisenhofer@uniklinikum-dresden.de (G.E.); 2 Core Unit for Molecular Tumor Diagnostics (CMTD), National Center for Tumor Diseases (NCT), 01307 Dresden, Germany; stephan.drukewitz@uniklinikum-dresden.de (S.D.); doreen.william@uniklinikum-dresden.de (D.W.); 3 German Cancer Consortium (DKTK), 01307 Dresden, Germany; 4 German Cancer Research Center (DKFZ), 69120 Heidelberg, Germany; 5 Institute of Clinical Chemistry and Laboratory Medicine, University Hospital Carl Gustav Carus, Technische Universität Dresden, Fetscherstrasse 74, 01307 Dresden, Germany; isabel.poser@uniklinikum-dresden.de (I.P.); susan.richter@uniklinikum-dresden.de (S.R.); markus.friedemann@uniklinikum-dresden.de (M.F.); 6 Institute for Diabetes and Cancer, Helmholtz Centre Munich, Ingolstaedter Landstr.1, 85764 Neuherberg, Germany; hermine.mohr@helmholtz-muenchen.de; 7 Joint Heidelberg-IDC Translational Diabetes Program, Heidelberg University Hospital, 69120 Heidelberg, Germany; 8 Department of Endocrinology, Diabetology and Clinical Nutrition, University Hospital Zurich (USZ), University of Zurich (UZH), 8091 Zurich, Switzerland; svenja.noelting@usz.ch; 9 Department of Medicine IV, University Hospital, LMU Munich, 80336 Munich, Germany; 10 Hereditary Endocrine Cancer Group, Spanish National Cancer Research Center, 28029 Madrid, Spain; mrobledo@cnio.es; 11 Centro de Investigación Biomédica en Red de Enfermedades Raras (CIBERER), 28029 Madrid, Spain; 12 German Institute of Human Nutrition Potsdam-Rehbruecke, Department of Experimental Diabetology, 14558 Nuthetal, Germany; 13 German Center for Diabetes Research (DZD), 85764 Neuherberg, Germany

**Keywords:** hypoxia resistance, pseudohypoxia, metastasis, pheochromocytoma, paraganglioma, drug resistance, epigenetic, hypermethylation

## Abstract

Continuous activation of hypoxia pathways in pheochromocytomas and paragangliomas (PPGLs) is associated with higher disease aggressiveness, for which effective treatment strategies are still missing. Most of the commonly used in vitro models lack characteristics of these pseudohypoxic tumors, including elevated expression of hypoxia-inducible factor (HIF) 2α. To address this shortcoming, we investigated whether recurrent hypoxia cycles lead to continuous activation of hypoxia pathways under normoxic conditions and whether this pseudohypoxia is associated with increased cellular aggressiveness. Rat pheochromocytoma cells (PC12) were incubated under hypoxia for 24 h every 3–4 days, up to 20 hypoxia–reoxygenation cycles, resulting in PC12 Z20 cells. PC12 Z20 control cells were obtained by synchronous cultivation under normoxia. RNA sequencing revealed upregulation of *HIF2α* in PC12 Z20 cells and a pseudohypoxic gene signature that overlapped with the gene signature of pseudohypoxic PPGLs. PC12 Z20 cells showed a higher growth rate, and the migration and adhesion capacity were significantly increased compared with control cells. Changes in global methylation, together with the pseudohypoxic conditions, may be responsible for the increased aggressiveness of this new model. The established sub-cell line with characteristics of pseudohypoxic PPGLs represent a complementary model for further investigations, for example, with regard to new therapeutic approaches.

## 1. Introduction

Pheochromocytomas and paragangliomas (PPGLs) are rare, endocrine tumors arising from neural crest-derived cells of the adrenal medulla or extra-adrenal paraganglia. PPGLs have a high degree of heritability. Germline or somatic mutations in one of over 20 driver genes can be identified in 70–80% of patients presenting with PPGL, and are associated with specific phenotypic features [1,2,3]. Based on this, PPGLs are derived into 2 main clusters, cluster 1 and cluster 2 PPGLs. Mutations in genes encoding the von Hippel–Lindau (*VHL*) tumor suppressor, succinate dehydrogenase subunits (*SDHx*), prolyl hydroxylase domain (*PHD1/PHD2*), fumarate hydratase (*FH*), malate dehydrogenase 2 (*MDH2*), mitochondrial 2-oxoglutarate/malate carrier (*SLC25A11*), isocitrate dehydrogenases (*IDH1/IDH2/IDH3B*), glutamic-oxaloacetic transaminase 2 (*GOT2*), dihydrolipoamide S-succinyltransferase (*DLST*), and hypoxia-inducible factor 2α (*HIF2α/EPAS1*) are characterized by an activation of hypoxia signaling pathways. The upregulation of hypoxia signaling pathways, normally activated in absence of oxygen (hypoxia), also occurs in PPGLs when oxygen is present (normoxia). This condition is termed pseudohypoxia and is a hallmark of cluster 1 PPGLs [4]. In addition, cluster 1 PPGLs are divided into 2 sub-clusters: cluster 1A includes PPGLs due to mutations in Krebs cycle related genes (*SDHx*, *FH*, *MDH2*, *SLC25A11*, *IDHs*, *GOT2*, *DLST*), while cluster 1B is related to mutations in genes of the hypoxia-signaling pathway (*VHL*, *PHDs*, *EPAS1*). Cluster 1 PPGLs and, in particular, PPGLs with mutations in *SDHB* are more prone to metastatic disease than PPGLs with mutations in genes that lead to an activation of kinase signaling pathways (cluster 2 PPGLs) [5]. Hypermethylation profiles, as well as alterations in fusion genes, are further associated with the occurrence of metastases in PPGLs [1]. Treatment options for metastatic PPGLs remain limited; hence, improved understanding of the molecular basis is required to identify novel therapeutic approaches.

Enhanced expression and stabilization of HIF2α (encoded by *EPAS1*) are specific characteristics of cluster 1 PPGLs that promote a pro-metastatic phenotype in these tumors [5,6,7,8,9]. In *SDHB*-mutated tumors, both HIF2α and ten eleven translocation (TET) dioxygenases-mediated hypermethylation synergistically drive the acquisition of metastatic properties [10]. This provides a rational for targeting HIF2α and DNA methylation in metastatic PPGLs, particularly for those with mutations in *SDHB*. Currently available preclinical in vitro models to evaluate such novel therapeutic approaches present with different limitations and often lack characteristic features of aggressive cluster 1 PPGLs [11,12]. Moreover, genetically engineered cell lines only reflect the changes associated with the particular mutation and do not allow for general conclusions about the increased aggressiveness of cluster 1 tumors and the associated therapeutic response.

In the present study, we therefore hypothesized that treatment of pheochromocytoma cells with recurrent hypoxia–reoxygenation cycles will lead to a constitutive activation of hypoxia signaling pathways in the obtained sub-cell line, modelling a pseudohypoxic phenotype in these cells that exhibits molecular and functional overlaps with cluster 1 PPGLs. In a proof-of-principle approach, we treated rat pheochromocytoma cells (PC12) with 10 or 20 hypoxia–reoxygenation cycles and characterized the resulting sub-cell lines in terms of growth characteristics and pro-metastatic behavior. In addition, RNA sequencing was used to investigate molecular overlaps with cluster 1 PPGLs. We further investigated whether changes in global DNA methylation are involved in generating specific phenotypic features in these cells.

## 2. Materials and Methods

**Figure 1:**
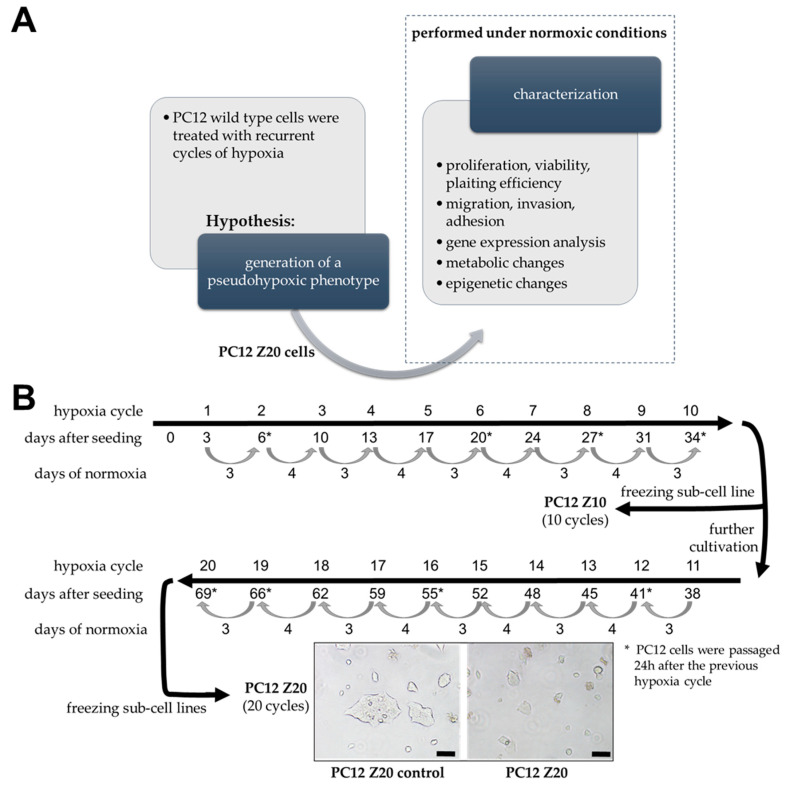
Schematic representation of the experimental procedure. (**A**) The experiments can be divided into two separate parts. First, generation of a sub-cell line by treatment of PC12 cells with recurrent cycles of hypoxia (hypoxia–reoxygenation cycles), based on the hypothesis that this procedure would lead to the generation of a pseudohypoxic phenotype in these cells. In the second part, cells were characterized under normoxic conditions to confirm the pseudohypoxic phenotype of the newly established sub-cell line. (**B**) Procedure to generate PC12 sub-cell lines. Cells were treated with recurring cycles of hypoxia (≤1% oxygen, 24 h hypoxia/cycle, followed by a reoxygenation phase of 3–4 days). After 20 (PC12 Z20) cycles, morphological changes were determined by phase contrast microcopy in comparison with control cells cultivated under normoxic conditions (PC12 Z20 control). Furthermore, sub-cell lines after 10 hypoxia–reoxygenation cycles were obtained and characterized under normoxic conditions. Scale bar: 100 µm.

Figure 1A shows an overview of the experimental procedure that build the basis for the present manuscript. Treatment with recurrent cycles of hypoxia (low oxygen; ≤1% oxygen), each followed by a reoxygenation phase (normoxic conditions), was initially used to generate new sub-cell lines that were subsequently characterized under normoxic conditions to confirm the hypothesis that treatment of pheochromocytoma cells with recurrent cycles of hypoxia leads to the formation of features of aggressive, pseudohypoxic cluster 1 PPGLs. The exact experimental details are described below.

Unless otherwise stated, all reagents and solutions were of the highest purity from Sigma-Aldrich (St. Louis, MO, USA). All cell culture media and additives were purchased from Gibco (Thermo Fisher Scientific, MA, USA), only fetal calf serum was obtained from Biowest (Riverside, CA, USA).

### 2.1. Cell Culture

Rat pheochromocytoma cell line, PC12 [13], and the human progenitor pheochromocytoma cell line, hPheo1 [14], were generous gifts from Arthur Tischler and Hans K. Ghayee. PC12 cells were cultivated in RPMI-1640 containing 10% horse serum (HS) and 5% fetal calf serum (FCS) on collagen A-coated cell culture dishes. For hPheo1 cells, RPMI-1640, containing 10% FCS and 2 mM Glutamax, was used (detailed experimental procedures and results are summarized in Supplementary Materials). If not stated otherwise, cells were cultivated under normoxic conditions (5% CO_2_, 37 °C, and 95% humidity) in a CO_2_ incubator (HERAcell, Thermo Scientific). For the simulation of hypoxic conditions, cells were incubated in reduced oxygen partial pressure (≤1% O_2_) using an incubator equipped with an oxygen-sensor (Gasboy, Labotect, Göttingen, Germany). All cells were passaged up to 10 times after thawing, and experiments were conducted after passaging the cells at least once. Therefore, cells were trypsinized (trypsin/EDTA; 0.05%/0.02%), diluted with complete medium and counted by using C-CHIPs (Neubauer improved). All cell lines and the subsequently described sub-cell lines were regularly tested to be mycoplasma free (MycoAlert Mycoplasma Detection Kit, Lonza, Basel, Switzerland).

### 2.2. Generation of Sub-Cell Lines by Treatment with Recurrent Hypoxia–Reoxygenation Cylces

To investigate the effects of recurrent cycles of hypoxia on the cellular and molecular behavior of PC12 cells, 1 × 10^6^ cells were seeded in 75 cm^2^ cell culture flasks in triplicate (10 cycles, 20 cycles, and normoxic control). After 3 days under normoxic conditions, medium was replaced and cells were incubated for 24 h under hypoxic conditions (O_2_ ≤ 1%). This procedure was repeated every 3–4 days (recurrent cycles of hypoxia) and in the meantime, cells were placed under normoxic conditions (reoxygenation). The procedure was repeated for 10 and 20 hypoxia–reoxygenation cycles (Figure 1B). An oxygen concentration of ≤1% was chosen because previous experiments with these cells were also performed by us at this oxygen level and we wanted to ensure comparability [15]. Upon reaching 90–100% confluence, cells were passaged 24 h after the last cycle of hypoxia. As control, an additional flask PC12 cells was treated as described, except that hypoxic conditions were replaced with normoxia. We generated 4 different sub-cell lines; PC12 Z10 and PC12 Z10 control cells, obtained after 10 cycles hypoxia or normoxia, as well as PC12 Z20 and PC12 Z20 control cells, collected after 20 cycles hypoxia or normoxia. When the cells reached 80–90% confluence after the last hypoxia cycle, they were frozen in Cryo-SFM freezing medium (PromoCell GmbH, Heidelberg, Germany) and stored in liquid nitrogen. For all experiments, cells were thawed and cultivated under normoxia at least for one passage. During the characterization of the cells, we observed that a stable phenotype was formed after four passages (data not shown; comparable effects before as well, but continuously increasing during the first three passages). Therefore, all experiments shown here were performed after at least four passages after thawing.

### 2.3. Viability Assay

Cell viability were analyzed using the CellTiter 96^®^ Aqueous One Solution Cell Proliferation Assay (Promega, Mannheim, Germany). In accordance to manufacturer’s instruction, cells (17.5 × 10^4^) were seeded in 96-well plates. After 48 h incubation, cells were incubated with CellTiter 96^®^ Aqueous One reagent (3.5 h) at 37 °C and the absorption was measured at 492 nm by Spark^®^ multimode microplate reader (Tecan Group Ltd., Männedorf, Switzerland).

### 2.4. Proliferation Assay

Cells (1.5 × 10^5^ per well) were seeded in 6-well plates and cultivated for 48 h, 72 h, or 144 h under normoxic or hypoxic conditions. After incubation, cells were washed with PBS, trypsinized, carefully resuspensed in medium and counted automatically using Tecan Cell Chips™ and the Spark^®^ multimode microplate reader (Tecan Group Ltd., Männedorf, Switzerland). Each well was counted in duplicate. For the calculation of the doubling time, www.doubling-time.com (last request: 29 November 2021) was used.

### 2.5. Clonogenic Cell Survival Assay

Clonogenic cell survival assays were used to determine the relative plating efficacy of the new sub-cell lines and their response towards chemotherapeutics. An optimized cell number of 500 cells per well were seeded in 6-well plates. After 24 h, cells were treated with cisplatin (10 µM), doxorubicin (1 µM), etoposide (5 µM), paclitaxel (10 µM), vincristine (10 µM), dacarbazine (10 µM), cyclophosphamine (10 µM), or DMSO, and cultured under normoxic conditions for 10 days. Cells were fixed and stained, as previously described [16]. Colonies including more than 50 cells were counted (Stemi 2000-c, Carl Zeiss AG, Oberkochen, Germany).

### 2.6. Migration Assay

The capacity for cells to migrate through 8 µm pores was determined by using TC-inserts (Sarstedt AG and Co. KG, Nümbrecht, Germany). All sub-cell lines (5 × 10^6^) were plated in 75 cm^2^ cell culture flask and cultivated for 24 h. Afterwards, cells were starved for 24 h using RPMI-1640 containing 0.2% bovine serum albumin (BSA), washed with PBS, trypsinized, and cell number was determined. As chemoattractant complete medium containing serum was used in each well of a 12-well plate. Cells (2 × 10^5^ cells/insert) were resuspended in RPMI-1640 (0.2% BSA) and added to the upper compartment of the TC-insert. After 24 h incubation, culture medium was replaced by RPMI-1640 (0.2% BSA) containing 1 µM calcein (BD^TM^ calcein AM Fluorescent Dye, BD Biosciences, Franklin Lakes, NJ, USA). After 1 h incubation under normoxic conditions, the lower compartment was washed with PBS and cells that migrated through the pores were trypsinized. Fluorescence intensity of calcein-stained cells was measured at 485_Ex_/528_Em_ using the Spark^®^ multimode microplate reader (Tecan Group Ltd., Männedorf, Switzerland).

### 2.7. Invasion Assay

To investigate the invasion capacity of the cells, TC-inserts were coated with Matrigel^®^ (BD Bioscience/RPMI-1640 + 0.2% BSA, 1/3, *v*/*v*). All following steps were conducted, analogously to the migration assay described above.

### 2.8. Adhesion Assay

The ability to adhere to the extracellular matrix proteins laminin and collagen was analyzed. Therefore, 1.5 × 10^6^ cells were plated in a cell culture flask and cultivated for 24 h (pre-culture). Cell culture plates (24-well) were coated with laminin (1:20 in PBS) or collagen A (1:25 in PBS) for 1 h at 37 °C. After incubation, coated plates were washed twice with PBS and unspecific binding sites were blocked with PBS containing 2% BSA for 1 h at 37 °C. Cells were washed 2 times with PBS, trypsinized, resuspended in RPMI-1640 containing 0.2% BSA, and seeded (1 × 10^5^ cells/well) in the laminin- or collagen-coated wells. After 30 min incubation at 37 °C, attached cells were stained and quantified as previously described by us [17].

### 2.9. Catecholamine Measurments

Cells (1.5 × 10^5^ per well) were seeded in 24-well plates and cultivated for 24 h under normoxic conditions. After the 24 h treatment, cells were washed with PBS, and incubated for 15 min in perchloric acid (0.4 M perchloric acid containing 0.5 mM ethylenediaminetetraacetic acid) on ice. The obtained cell extracts were centrifuged and supernatants were analyzed for catecholamines by liquid chromatography with electrochemical detection, as described previously [6]. Concentrations of catecholamines were calculated relative to total number of cells/well. For cell counting, cells of an additional well were trypsinized, carefully resuspensed, and counted in duplicate by using C-CHIPs (Neubauer improved).

### 2.10. TCA Cycle Metabolites

Cells (2.5 × 10^5^ per well) were seeded in 6-well plates. After 48 h, plates were stored on ice, medium was removed, and cells were washed 4 times with PBS. Cells were extracted with 100% cold MeOH. Extracts were transferred to Eppendorf tubes, centrifuged (16,000× *g*, 5 min, 4 °C), and supernatants were dried using a speed vac concentrator (Thermo Scientific) and stored at −80 °C. TCA cycle metabolites were analyzed by UHPLC-MS/MS as previously describe [18].

### 2.11. TET Activity Assay

Cells (1.5 × 10^6^) were seeded in 75 cm^2^ cell culture flasks. After 24 h, cells were treated with 0.5 mM ascorbic acid, 10 µM 5-octyl-alpha-ketoglutarate (membrane permeable α-ketoglutarate) or DMSO as control and incubated for 48 h. The medium was removed and cells were washed with PBS before being detached with a cell scraper. Cells were counted and after a centrifugation step, supernatants were removed. Nuclear proteins were isolated from the obtained pellets using the Nuclear Extraction Kit (Abcam plc., Cambridge, UK, ab113474). The obtained extracts (50 µg/sample) were used afterwards to determine the TET activity by performing TET Hydroxylase Activity Quantification Kit (Fluorometric) (abcam, ab156913) as described by the manufacturer.

### 2.12. Global Methylation Assay

Cells (0.5 × 10^6^) were seeded in 25 cm^2^ cell culture flasks. After 24 h, cells were treated with 0.5 mM ascorbic acid, 10 µM 5-octyl-alpha-ketoglutarate, or DMSO, as control, and incubated for 48 h. Cells were washed with PBS, detached with trypsin, and counted. Cells (1 × 10^6^) were transferred to Falcon tubes, centrifuged, and pellets were washed with ice-cold PBS. DNA was isolated using Quick-DNA Microprep Kit (Zymo Research, Irvine, CA, USA, #D3020). For the determination of global DNA methylation (5-methylcystosine/total DNA), 100 ng isolated DNA was analyzed using the Global DNA Methylation Assay Kit (abcam, ab233486).

### 2.13. Pre-Treatment with Ascorbic Acid or 5-Octyl-alpha-ketoglutarate

To investigate whether changes in methylation are involved in the pro-metastatic properties of PC12 Z20 cells, treatment with 0.5 mM ascorbic acid or 10 µM 5-octyl-alpha-ketoglutarate was performed to promote TET-dependent demethylation. To determine the effect on cell growth, cells were treated with ascorbic acid or 5-octyl-alpha-ketoglutarate, and cell numbers were determined after 240 h after treatment, as described above. Moreover, we pretreated the cells with ascorbic acid or 5-octyl-alpha-ketoglutarate for 6 days (pre-culture) before performing migration, invasion, and adhesion assays, as described above.

### 2.14. RNA Isolation and qRT-PCR

RNA was isolated from cell pellets using NucleoSpin RNA Plus (Machery-Nagel GmbH, Düren, Germany) as described in manufacturer’s instructions. Reverse transcription of RNA, qRT-PCR, and primer pairs were described previously by us [15].

### 2.15. RNA Sequencing

For library preparation, TruSeq Stranded mRNA Library Prep Kit (Illumina Inc, San Diego, CA, USA) according to the manufacturer’s protocol was used, starting with 1 μg total RNA. All barcoded libraries were pooled and sequenced 2 × 75 bp paired-end on an Illumina NextSeq500 platform to obtain a minimum of 10 × 10^6^ reads per sample. The data obtained were made publicly available (BioProject: PRJNA785777; ‘sample_control’ corresponds to PC12 Z20 control; ‘sample’ corresponds to PC12 Z20; data sets marked ‘normoxia’ were used here).

### 2.16. Bioinformatics Analysis

Within the framework of the bioinformatic workflow, raw reads were trimmed using trimmomatic [19] and aligned using STAR [20], Rnor_6.0.102 was used as reference genome. Read counts were extracted from the alignments using the featureCounts method of the subread package [21]; afterwards, DESeq2 was applied to identify differentially expressed genes [22]. Only genes with multiple testing adjusted *p*-values (padj from DESeq2) < 0.05 were considered significant. All pathway analysis was performed using the gseapy package [23,24]. Gene symbols of significant upregulated genes from PC12 Z20 were extracted and compared to significant upregulated genes from the cluster 1 (C1) PPGLs vs. cluster 2 (C2) PPGLs using gene expression array datasets of 91 PPGLs [8,25]. Extraction of mRNA data, background correction, and normalization was performed as previously described [5]. Overlapping genes between the two different data sets were extracted and fed into an EnrichR analyses to identify enriched KEGG pathways in the overlap of C1 PPGLs and PC12 Z20 [24,26].

### 2.17. Statistical Analysis

Descriptive data were expressed as means ± standard error of means (SEM). Statistical analyses considered the number (*n*) of technical and biological replicates within independent experiments. Statistical analyses were carried out by Sigma Plot 12.5 (Systat Software GmbH, Erkrath, Germany). After confirmation of normal distribution by Shapiro–Wilk test, one-way analysis of variance (ANOVA) with post hoc Bonferroni tests (more than two means) or unpaired *t*-test (two means) were performed.

## 3. Results

### 3.1. Recurrent Cycles of Hypoxia Lead to the Establishment of a Pro-Metastatic Phenotype with Enhanced Growth Characteristic under Normoxic Conditions

**Figure 2:**
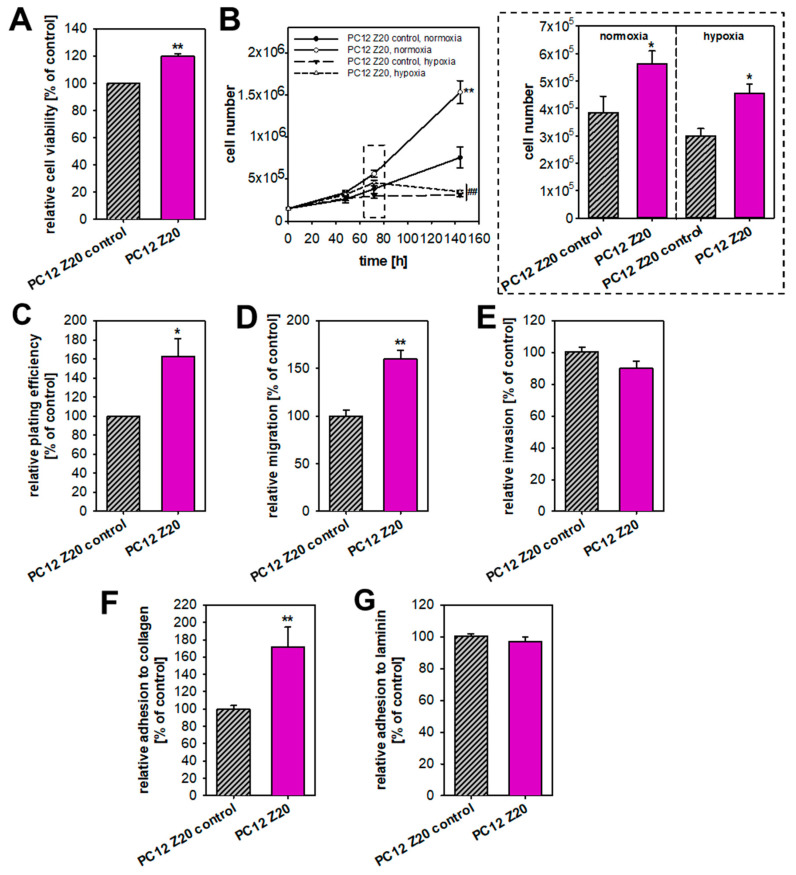
PC12 sub-cell line obtained after 20 hypoxia–reoxygenation cycles revealed enhanced proliferative and pro-metastatic behavior under normoxic conditions compared with control cells. (**A**) PC12 Z20 cells showed an increased cell viability compared with PC12 Z20 control cells. Twelve independent experiments (*n* = 36). (**B**) The growth rate of PC12 Z20 cells was significantly higher under normoxic conditions and to some extent also under hypoxic conditions compared with control cells. A total of six independent experiments (*n* = 12). (**C**) Clonogenic survival assays revealed an enhanced plating efficiency of PC12 Z20 cells. A total of three independent experiments (*n* = 3). The (**D**) migration capacity of the PC12 Z20 cells was increased, while the (**E**) invasion capacity was not affected. From five to six independent experiments (*n* = 32–36). PC12 Z20 cells showed a diminished adhesion to (**F**) collagen and in trend to (**G**) laminin compared with the control cells. A total of four independent experiments (*n* = 16). Mean ± SEM; unpaired *t*-test for the comparison of two groups or Anova and Bonferroni post hoc test for more than two groups; comparison vs. PC12 Z20 control * *p* < 0.05, ** *p* < 0.001; comparison vs. normoxic conditions ^##^ *p* < 0.001.

Pseudohypoxic PPGLs are more prone to develop metastatic disease [5], but available in vitro models often lack typical characteristics of a pseudohypoxic phenotype, such as an immature catecholamine phenotype, increased pro-metastatic behavior, and upregulation of hypoxia-related genes under normoxic conditions [11]. Based on the hypothesis that recurrent cycles of extrinsic hypoxia (≤1% oxygen) leads to a manifestation of a pseudohypoxic phenotype, we established PC12 sub-cell lines using repeated exposure to hypoxia–reoxygenation cycles. Between the 24 h hypoxia cycles, cells were cultured for 3–4 days under normoxic condition, for reoxygenation. This was necessary because we previously showed that PC12 go into a growth arrest under longer periods of hypoxia [16]. The oxygen concentration of ≤1% was chosen to achieve comparability with our previous studies [15]. In parallel, cells were synchronously cultured under normoxic conditions. These control cells were treated in exactly the same way, except that they remained under normoxia. After 10 and 20 cycles of hypoxia or normoxia, respective sub-cultures of the hypoxia-exposed cells (PC12 Z10 and PC12 Z20) and the corresponding control cells (PC12 Z10 control and PC12 Z20 control) were obtained and characterized. PC12 Z20 cells lost their roundish shape and appeared more flat, while PC12 Z10 cells showed no clear phenotypic change (Figure 1B and Figure S1A). PC12 Z20 cells displayed significantly altered proliferative properties (Figure 2). The viability, as well as the growth rate, of PC12 Z20 cells (doubling time = 43.0 h) was significantly increased compared with PC12 Z20 control cells (doubling time = 61.8 h) (Figure 2A,B). Under hypoxic conditions, PC12 Z20 cells showed increased cell numbers after 72 h, but similar to control cells, PC12 Z20 cells stop growing after prolonged exposure to hypoxic conditions (Figure 2B). Clonogenic survival assays revealed a significantly enhanced plating efficiency of PC12 Z20 compared with control cells. In contrast, PC12 Z10 showed only a limited alteration in their growth characteristics compared with the PC12 Z10 control cells (Figure S1B–D). We therefore decided to focus on the PC12 Z20 cells and the corresponding control cells for further characterization.

During the invasion–metastasis cascade, tumor cells are subject to various cellular changes that alter the behavior of the cells. Hypoxic and pseudohypoxic conditions can favor a pro-metastatic phenotype by induction of a neuroendocrine–mesenchymal transition (neuroendoMT) [5,9,10]. Therefore, we investigated the pro-metastatic behavior of the two PC12 Z20 sub-cell lines. The migration capacity of the PC12 Z20 cells was significantly enhanced (Figure 2D), while the invasion capacity was not affected compared to the PC12 Z20 control cells (Figure 2E). Moreover, PC12 Z20 cells showed an increased adhesion to collagen compared with the control cells (Figure 2F). No differences in the adhesion capacity to laminin were observed (Figure 2G). In summary, PC12 Z20 cells treated with recurrent cycles of hypoxia showed enhanced growth characteristics and a pro-metastatic phenotype under normoxic conditions.

To confirm that recurrent cycles of hypoxia are suitable to induce a pseudohypoxic phenotype, we performed comparable experiments with human chromaffin progenitor cells isolated from a pheochromocytoma (hPheo1 [14], Supplementary Materials). In this significantly more proliferating cell line, 10 hypoxia cycles were sufficient for the induction of pro-metastatic properties (Figures S2 and S3).

Tumor hypoxia is known to contribute to an overall diminished efficiency of chemotherapy [27]. Based on this, we aimed to clarify whether the cells treated with recurrent hypoxia cycles show resistance to common chemotherapies and selected target drugs compared to the control cells. Both PC12 Z20 and hPheo1 Z10 cells showed no difference in their response towards treatment with vincristine, etoposide, cisplatin, decarbazine, cyclophosphamine, paclitaxel, or doxorubicine, compared to the corresponding control cells (Supplementary Materials, Tables S1 and S2). We were previously able to demonstrate synergistic effects of the targeted combination therapy using the specific phosphatidylinositol-3-kinase α inhibitor BYL719 together with the mammalian target of rapamycin inhibitor everolimus on pheochromocytoma cell spheroids and primary cultures of human pheochromocytomas [28]. hPheo1 Z10 spheroids showed resistance to combination treatment compared with control spheroids, whereas no differences between spheroids were observed for single treatment with either everolimus or BYL719 alone (Supplementary Materials S1, Figure S4). Regardless of resistance, the synergistic effect of this combination therapy was also demonstrated in this spheroid model, confirming previous results [28].

### 3.2. Gene Expression Analysis Revealed a Pseudohypoxic Gene Signature in PC12 Z20 Cells Compared with the Control

For further characterization purposes, we performed RNA sequencing and compared the mRNA expression of PC12 Z20 and PC12 Z20 control cells under normoxic conditions. The 20 most up- and downregulated genes of this comparison are summarized in the Supplementary Materials (Supplementary Materials S1, Table S3). PC12 Z20 cells showed a significant upregulation of *Epas1* expression (log2 fold change = 0.457, *p* = 2.44 × 10^−5^), while *Hif1α* was not affected. This is in line with the situation in PPGLs, where mutations in cluster 1 genes lead to a pseudohypoxic phenotype characterized by an enhanced expression and stabilization of HIF2α, while HIF1α remains unaffected [6,7,8,25]. In addition, the hypoxia marker carboanhydrase IX (*Ca9*) [29] showed a significant upregulation in PC12 Z20 cells (log2 fold change = 1.601, *p* < 0.001). Nevertheless, stabilization of HIF2α was not affected (Figure S5).

**Figure 3:**
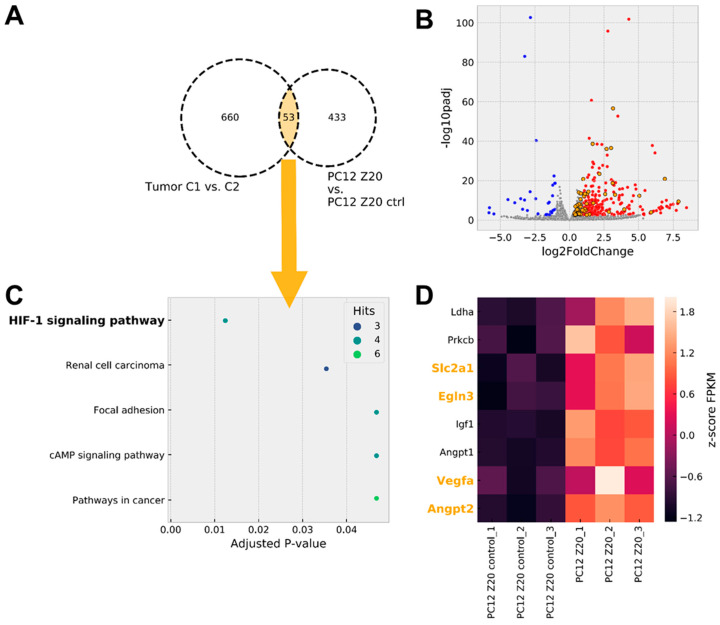
Pseudohypoxic gene expression signature in PC12 Z20 cells cultivated under normoxic conditions. (**A**) Microarray data including tumor tissue of pseudohypoxic cluster 1 PPGLs (C1, include tumors with *VHL*, *EPAS1*, and *SDHx* mutations) and cluster 2 PPGLs (C2, include tumors with *NF1*, *RET*, *MAX*, or *TMEM127* mutations leading to an activation of kinase signaling pathways) showed an upregulation of 660 genes in C1 compared with C2 PPGLs. RNA sequencing of PC12 Z20 control and PC12 Z20 cells revealed a regulation of 433 genes in the PC12 Z20 cells. An overlap of 53 genes between both datasets (C1 PPGLs vs. C2 PPGLs and PC12 Z20 vs. PC12 Z20 control) indicate similarities between the pseudohypoxic cluster 1 PPGLs (C1) and PC12 Z20 cells compared to the respective cluster 2 PPGLs (C2) and PC12 Z20 control cells (Supplementary Materials S2). (**B**) Volcano plot of genes differentially expressed in PC12 Z20 control vs. PC12 Z20 cells (Supplementary Materials S3 and S4). Genes overlapping with pseudohypoxic C1 PPGLs are highlighted in orange. (**C**) EnrichR analysis of genes overlapping between pseudohypoxic C1 and PC12 Z20 cells revealed the HIF1 signaling pathway as mostly regulated KEGG pathway. (**D**) Heat map of genes significantly regulated in HIF1 signaling pathway in PC12 Z20 cells compared with PC12 Z20 control cells. Genes overlapping with pseudohypoxic C1 PPGLs are highlighted in orange.

To further characterize the extent to which PC12 Z20 cells, cultivated under normoxic conditions, reflect the situation of pseudohypoxic cluster 1 PPGLs, gene expression profiles of PC12 Z20 cells were compared with available microarray data [8] from PPGL tumor tissue (Figure 3, Supplementary Materials S3 and S4). Tissue of cluster 1 PPGLs (tumors with *VHL*, *EPAS1*, and *SDHx* mutations) showed a significant upregulation of 660 genes compared with cluster 2 PPGLs (tumors with *NF1*, *RET*, *MAX,* or *TMEM127* mutations). Our RNA sequencing data revealed a significant upregulation of 433 genes in PC12 Z20 compared with control cells. An overlap of 53 genes between both comparisons indicate similarities between the pseudohypoxic cluster 1 PPGLs and PC12 Z20 cells in the upregulation of specific genes (Figure 3A,B, Supplementary Materials S2). Gene set enrichment analysis of genes overlapping between cluster 1 PPGLs and PC12 Z20 cells revealed that the HIF1 signaling pathway is the most regulated KEGG pathway within the overlapping genes (Figure 3C). Most of the HIF1α target genes are also transcriptionally activated by HIF2α, which appears to be the case for both cluster 1 PPGLs and PC12 Z20 cells based on increased *EPAS1* expression. Further enrichment of genes were found in the KEGG pathways related to renal cell carcinoma, focal adhesion, cAMP signaling, and pathways in cancer. Focal adhesion, cAMP signaling pathways, and pathways in cancer were also under the most upregulated KEGG pathways in PPGLs with *EPAS1* mutation compared with tumors bearing a mutation in one of the cluster 2 susceptibility genes (*NF1*, *RET*, *MAX*) [5]. Besides *Scl2a1*, *Egln3*, *Vegfa*, and *Angpt2*, four additional genes of the HIF signaling pathway (*Ldha*, *Prkcb*, *Igf1*, and *Angpt1*) were upregulated in PC12 Z20 cells compared with the control cells (Figure 3D).

### 3.3. PC12 Z20 Cells Showed an Accumulation of TCA Cycle Metabolites and a Decrease in the Cellular Catecholamine Content

**Figure 4:**
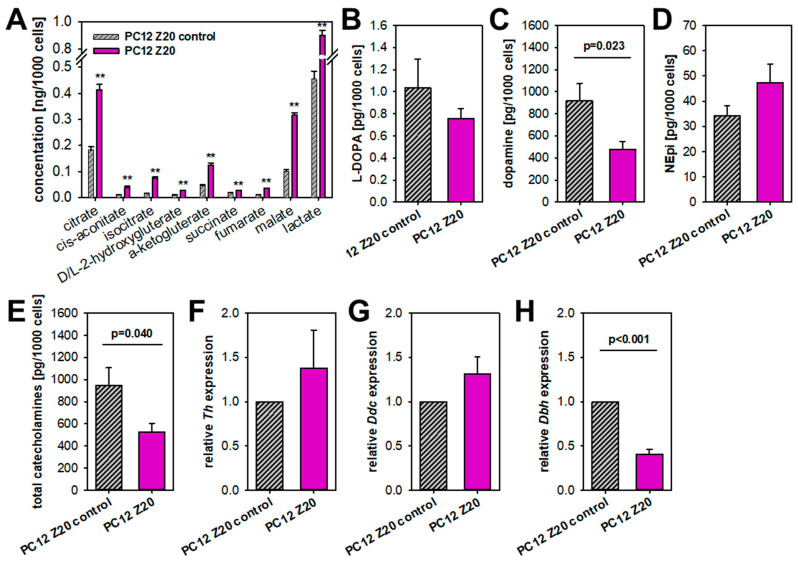
Metabolic differences between PC12 Z20 and PC12 Z20 control cells under normoxic conditions. (**A**) PC12 Z20 cells showed an accumulation of TCA cycle metabolites compared with the control cells. Four independent experiments (*n* = 16). Mean ± SEM; Anova and Bonferroni post hoc test comparison vs. PC12 Z20 control ** *p* < 0.001. (**B**) L-DOPA, (**C**) dopamine, (**D**) norepinephrine (NEpi) and (**E**) total catecholamine content of PC12 Z20 and PC12 Z20 control cells. Four independent experiments (*n* = 8). Relative expression of (**F**) tyrosine hydroxylase (*Th*), (**G**) DOPA decarboxylase (*Ddc*), and (**H**) dopamine beta-hydroxylase (*Dbh*) in PC12 Z20 and PC12 Z20 control cells. Mean ± SEM; unpaired *t*-test comparison vs. PC12 Z20 control.

Especially, PPGLs of the TCA cycle-related cluster 1A are known to show profound changes in central carbon metabolism with accumulation of oncometabolites, such as succinate, fumarate, and 2-hydroxygluterate, which subsequently lead to an inhibition of α-ketogluterate-dependent dioxygenases, including PHDs and TET dioxygenases [4]. We therefore investigated TCA cycle metabolites in both sub-cell lines (Figure 4A). All measured TCA cycle metabolites and lactate were significantly elevated in PC12 Z20 cells compared with PC12 Z20 control cells (1.98–4.4 fold change), indicating a higher metabolic activity of these cells. An accumulation of one specific metabolite in high concentrations was not observed.

Pseudohypoxic PPGLs are furthermore associated with an immature catecholamine phenotype, characterized by the absence of PNMT, low tissue catecholamine contents, and higher catecholamine secretion in contrast to cluster 2 PPGLs [30]. We therefore investigated the cellular catecholamine content of our new sub-cell lines (Figure 4B–E). Dopamine levels were significantly diminished in PC12 Z20 cells (Figure 4C), while its precursor L-DOPA was only slightly reduced compared with the control cells (Figure 4B). Both sub-cell lines showed comparable norepinephrine levels (Figure 4D). Overall, PC12 Z20 cells revealed a decrease in the cellular catecholamine levels compared with the control cells (Figure 4E). On gene level, only a downregulation of dopamine beta-hydroxylase (*Dbh*), which converts dopamine to norepinephrine, was observed, while the expression of tyrosine hydroxylase (*Th*) and DOPA decarboxylase (*Ddc*) was not affected (Figure 4F–H). Our data indicate reduced catecholamine biosynthesis/storage in PC12 Z20 cells, which are characterized by significant lower total catecholamine content compared with PC12 Z20 control cells.

### 3.4. Epigenetic Changes in PC12 Z20 Cells

**Figure 5:**
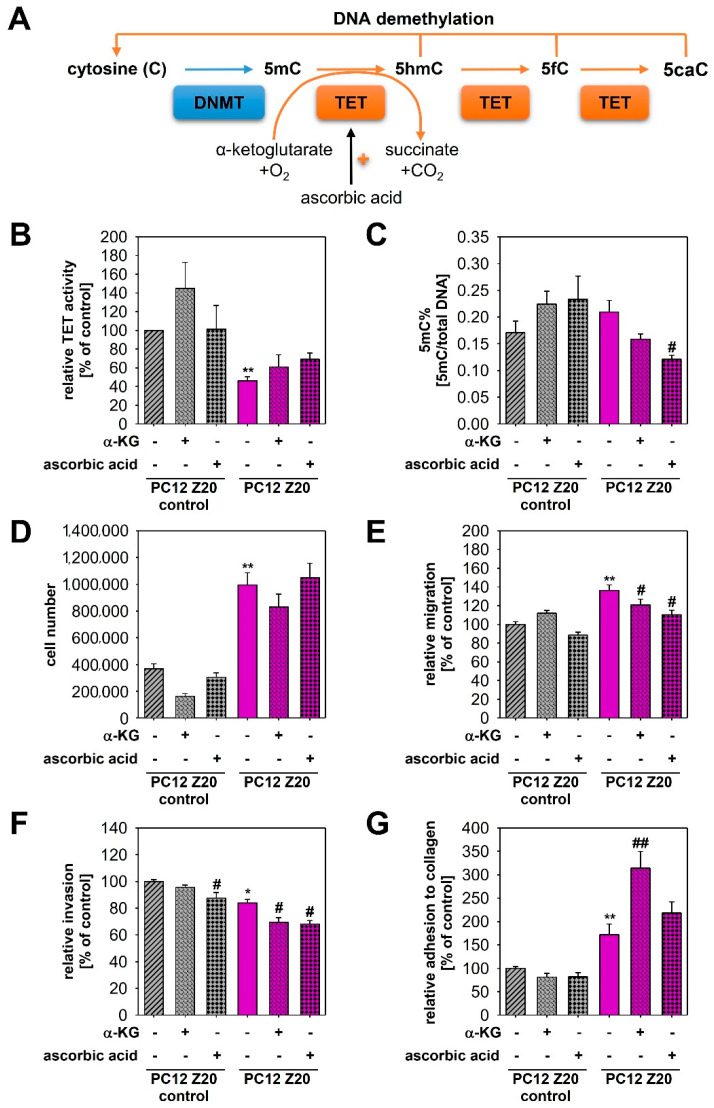
Epigenetic changes in PC12 Z20 cells and their phenotypic consequences. (**A**) DNA methylation is exerted by DNA methyltransferases (DNMTs), which transfer methyl groups from S-adenosylmethionine to cytosine (C) forming 5-methylcystosine (5mC). Demethylation is initiated by α-ketoglutarate (α-KG)-dependent ten eleven translocation (TET) dioxygenases that catalyze the oxidation of the methyl groups thereby converting 5mC to 5-hydroxymethylcytosine (5hmC), 5-formylcytosine (5fC), and 5-carboxycytosine (5caC). In addition to α-KG, ascorbic acid can also increase the activity of TETs. (**B**) PC12 Z20 cells showed a significant downregulation of TET enzyme activity compared with PC12 Z20 control cells. Treatment with either 10 µM α-KG or 0.5 mM ascorbic acid resulted in a slight upregulation of the TET activity in PC12 Z20 cells. A total of three independent experiments (*n* = 5). (**C**) Global DNA methylation assays revealed in trend an upregulation of 5mC in PC12 Z20 cells compared with control that could be diminished by treatment with ascorbic acid or α-KG. (**D**) Cell number after 240 h cultivation in absence or presence of either α-KG or ascorbic acid. A total of four independent experiments (*n* = 8). Influence of α-KG and ascorbic acid on (**E**) migration, (**F**) invasion, and (**G**) adhesion capacity of PC12 Z20 and PC12 Z20 control cells. Between three and four independent experiments (*n* = 16–18). Mean ± SEM; Anova and Bonferroni post hoc test comparison vs. PC12 Z20 control * *p* < 0.05, ** *p* < 0.001; vs. untreated respective sub-cell line ^#^ *p* < 0.05, ^##^ *p* < 0.001.

PPGLs often carry epigenetic alterations [31]. In particular, aberrant DNA methylation has been described as an important factor for the establishment of a pro-metastatic phenotype in *SDHB*-mutated tumors, whereby additional activation of HIF2α seems to be required for the manifestation of an aggressive phenotype [10]. Mechanistically, accumulation of oncometabolites (e.g., succinate and fumarate) due to mutations in *SDHx* or *FH* inhibits α-ketoglutarate-dependent TETs that hydroxylate methylated cytosine (5-methylcytosine—5mC) into hydroxymethylcytosine (5hmC) and thereby compromise DNA demethylation (Figure 5A). The profound alterations of TCA cycle metabolites in PC12 Z20 cells (Figure 4B) and the observation that a stable phenotype occurred only four to five passages after the last hypoxia cycle suggests the involvement of epigenetic mechanisms. We therefore investigated TET-mediated effects in our newly established sub-cell lines. PC12 Z20 cells revealed a significant downregulation of TET enzyme activity in comparison with PC12 Z20 control cells (Figure 5B). Treatment with ascorbic acid or a membrane-permeable α-ketoglutarate derivate, 5-octyl-alpha-ketoglutarate, led in trend to a reanimation of TET enzyme activity in PC12 Z20. Next, we examined global DNA methylation (5-methylcystosine/total DNA). The 5mC levels were slightly higher in PC12 Z20 cells compared with control and were significantly reduced by treatment with ascorbic acid (Figure 5C).

To clarify whether the decreased TET activity may contribute to the formation of the pro-metastatic phenotype of PC12 Z20 cells, we treated the cells for 5 days with ascorbic acid or 5-octyl-alpha-ketoglutarate and characterized the impact on proliferative and pro-metastatic properties of these cells (Figure 5D–G). Treatment with ascorbic acid or α-ketoglutarate showed no effect on the cell number (Figure 5D), but reduced significantly the migration capacity of PC12 Z20 cells, while control cells were not affected (Figure 5E). Moreover, invasion capacity of PC12 Z20 cells was significantly reduced by the treatment with ascorbic acid or α-ketoglutarate (Figure 5F). Ascorbic acid also reduced the invasion capacity of PC12 Z20 control cells. The adhesion to collagen was further enhanced by the treatment with α-ketoglutarate (Figure 5G). These data indicated that epigenetic changes may contribute to the manifestation of the specific phenotype in PC12 Z20 cells generated by treatment with recurrent hypoxia–reoxygenation cycles.

## 4. Discussion

**Figure 6:**
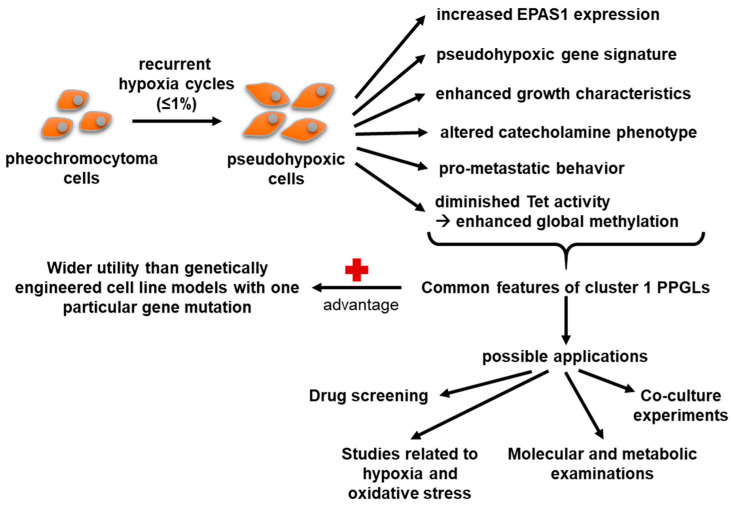
Characteristics and possible applications of the new established PC12 sub-cell lines.

Constitutive activation of hypoxia signalling pathways is a common feature of aggressive cluster 1 PPGLs. Through the treatment of pheochromocytoma cells with recurrent cycles of hypoxia, we established a new pheochromocytoma sub-cell line that exhibits enhanced pseudohypoxic properties, including a pro-metastatic phenotype, as well as a pseudohypoxic gene signature. The identified overlaps with pseudohypoxic cluster 1 PPGLs make PC12 Z20 cells and their respective control cells a suitable model for preclinical studies. The new cell model does not only reflect specific features of a particular cluster 1 PPGL driver mutation, but rather combines common features of the entire cluster, thus complementing the already available cell line models. It therefore represents a suitable model for further investigations, for example, with regard to new therapeutic approaches (Figure 6).

In analogy to experimental approaches for generation of radiation- or chemotherapy-resistant cell lines, for which recurrent cycles of irradiation or chemotherapeutics are applied [32,33,34], we followed a similar strategy using recurrent cycles of extrinsic hypoxia. PC12 cells, as well as the mouse pheochromocytoma cells—MPC and MTT—show growth arrest under hypoxic conditions (O_2_ ≤ 1%) [17]. The new established sub-cell lines do not overcome this phenotype. Nevertheless, we showed that recurrent hypoxia cycles activate hypoxia signaling pathways under normoxic conditions, which remained stably activated over several passages. Comparable with the molecular situation in cluster 1 PPGLs, only expression of *Epas1* was upregulated in our cell line model, while expression of *Hif1α* remained similar to control cells. This can be explained by temporal differences in the response of HIFαs to hypoxia. HIF1α is predominantly stabilized as an acute response to severe hypoxia, whereas HIF2α mediates the chronic hypoxia response and is stabilized in milder hypoxia [35]. Overlaps with the pseudohypoxic gene signature of cluster 1 PPGLs and the upregulation of genes involved in the HIF1α signaling pathway confirmed the constitutive activation of hypoxia signaling in these cells.

Consistent with our previous findings that HIF2α promoted a pro-metastatic phenotype in pheochromocytoma cells [5], activation of hypoxia signaling pathways through enhanced expression of *Epas1* also led to enhanced pro-metastatic properties in our novel sub-cell lines. This is line with other studies that indicate a potentially crucial role of cyclic hypoxia in driving tumor aggressiveness [36]. In addition, decreased TET activity leading to slightly increased global methylation indicates that epigenetic changes may contribute to the pro-metastatic phenotype of these cells. Morin et al. revealed synergistic effects of TET repression and pseudohypoxia, in particular characterized by enhanced activation of HIF2α, in the acquisition of a pro-metastatic phenotype [10]. Our data suggest that treatment with ascorbic acid or α-ketoglutarate slightly increased TET activity and thereby decreased global DNA methylation. This treatment also partially reduced the pro-metastatic properties of PC12 Z20 cells. However, ascorbic acid also influences redox homeostasis. In pheochromocytoma cells with low expression of SDHB, pseudohypoxia is associated with iron accumulation, which contributes to elevated oxidative stress in these cells [37]. Targeting redox homeostasis with ascorbic acid showed promise as therapeutic strategy for *SDHB*-mutated PPGLs [37]. Treatment with α-ketoglutarate also affects processes other than TET activity, since α-ketoglutarate-dependent enzymes have a variety of functions that may be involved in the response to α-ketoglutarate treatment. These mechanisms also likely contribute to the observed effects and may explain the difference in efficacy between the various assays we used.

Tumor hypoxia is associated with increased resistance to various therapeutic approaches, including radiotherapy and chemotherapy [38]. In monolayer culture, no differences in the response to several chemotherapeutic agents were observed. The tumor microenvironment is a critical driver of therapy resistance [39]. The better reflection of the tumor microenvironment, for example through enhanced cell–cell interactions and the formation of nutrient and oxygen gradients, may be an explanation why spheroids are better suited as a model to investigate therapy resistance. We have previously shown that Hif2α-expressing pseudohypoxic spheroids of pheochromocytoma cells (MPC) exhibit increased resistance to beta particle-emitting [^177^Lu]LuCl_3_ and external X-ray irradiation compared with Hif2α-deficient controls [40]. Moreover, hPheo1 Z10 spheroids showed resistance towards treatment with everolimus and BYL719 compared with the control spheroids. Importantly, the synergistic effect of this targeted combination therapy persists and thereby confirm our previous results [28].

For the establishment of pseudohypoxic cell models using a similar approach to ours, the following points should be considered. Phenotypic changes in PC12 and hPheo1 cells were dependent on the number of hypoxia–reoxygenation cycles, whereby changes seem to occur more rapidly in faster proliferating cells (doubling time hPheo1: 32.2 h; doubling time PC12: 61.8 h). Therefore, it is critical to study different numbers of hypoxia–reoxygenation cycles. For this purpose, we collected RNA and protein samples from each passage (Figure 1) to observe molecular changes during progression (data not shown).

## 5. Conclusions

In the proof-of-principle approach used in the present study, treatment of pheochromocytoma cells with recurrent cycles of hypoxia resulted in the establishment of new PC12 sub-cell lines with characteristics of pseudohypoxic cluster 1 PPGLs, including increased growth characteristics, a pro-metastatic behavior, pseudohypoxic gene expression profile, and epigenetic changes. Thus, the new sub-cell lines represent a suitable model for further investigations, for example, with regard to new therapeutic approaches that complement the existing in vitro PPGLs models.

## Data Availability

The data presented in this study are available in Supporting information S3 and S4.

## Supplementary Materials

The following Supplementary Materials can be downloaded at: https://www.mdpi.com/article/10.3390/cells11030560/s1, Supporting information S1 contain Figure S1: Impact of ten recurrent cycles of hypoxia on the growth characteristics of PC12 cells, Figure S2: Schematic representation of the experimental procedure and the time processes during generation of new hPheo1 sub-cell lines, Figure S3: Impact of recurrent cycles of hypoxia on the pro-metastatic behavior of hPheo1 cells, Figure S4: Therapy response to targeted therapies of hPheo1 Z10 spheroids and their control cells, Figure S5: HIF2α expression in PC12 Z20 and PC12 Z20 control cells, Table S1: Impact of common chemotherapeutics on the viability of PC12 Z20 and PC12 Z20 control cells, Table S2: Impact of common chemotherapeutics on viability of hPheo1 Z10 and hPheo1 Z10 control cells, Table S3: Twenty most up- or downregulated genes identified via RNA sequencing in PC12 Z20 cells vs. PC12 control. Supporting information S2: Genes overlapping between pseudohypoxic cluster 1 PPGLs and PC12 Z20 cells compared to the respective cluster 2 PPGLs and PC12 Z20 control cells. Supporting information S3: Genes downregulated in PC12 Z20 cells compared with the PC12 Z20 control cells. Supporting information S4: Genes upregulated in PC12 Z20 cells compared with the PC12 Z20 control cells.
