# Supplementary material for: Treatment of Pheochromocytoma Cells with Recurrent Cycles of Hypoxia: A New Pseudohypoxic In Vitro Model
Source: Cells. 2022 Feb 5;11(3):560. doi: 10.3390/cells11030560 (PMC8834104; doi:10.3390/cells11030560)
Supplement: Supplementary file 1 [file cells-11-00560-s001.zip › Supporting information_1_Rev copy.pdf]

**Supporting information**

# Treatment of pheochromocytoma cells with recurrent cycles of hypoxia: A new pseudohypoxic in vitro model

**Jana Helm[1], Stephan Drukewitz[2,3,4], Isabel Poser[5], Susan Richter[5], Markus Friedemann[5], Doreen William[2,3,4], Hermine Mohr[6,7], Svenja Nölting[8,9], Mercedes Robledo[10,11], Stefan R. Bornstein [1], Graeme Eisenhofer [1,5] and Nicole Bechmann [1,5,12,13]***

[1] Department of Medicine III, University Hospital Carl Gustav Carus, Technische Universität Dresden, Fetscherstrasse 74, 01307 Dresden, Germany.

[2] Core Unit for Molecular Tumor Diagnostics (CMTD), National Center for Tumor Diseases (NCT), 01307 Dresden, Germany

[3] German Cancer Consortium (DKTK), 01307 Dresden, Germany

[4] German Cancer Research Center (DKFZ), 69120 Heidelberg, Germany

[5] Institute of Clinical Chemistry and Laboratory Medicine, University Hospital Carl Gustav Carus, Technische Universität Dresden, Fetscherstrasse 74, 01307 Dresden, Germany.

[6] Institute for Diabetes and Cancer, Helmholtz Centre Munich, Ingolstaedter Landstr.1, 85764 Neuherberg, Germany

[7] Joint Heidelberg-IDC Translational Diabetes Program, Heidelberg University Hospital, 69120 Heidelberg, Germany

[8] Department of Endocrinology, Diabetology and Clinical Nutrition, University Hospital Zurich (USZ) and University of Zurich (UZH), Zurich, Switzerland

[9] Department of Medicine IV, University Hospital, LMU Munich, Munich, Germany

[10] Hereditary Endocrine Cancer Group, Spanish National Cancer Research Center, Madrid, Spain

[11] Centro de Investigación Biomédica en Red de Enfermedades Raras (CIBERER), Madrid, Spain

[12] German Institute of Human Nutrition Potsdam-Rehbruecke, Department of Experimental Diabetology, 14558 Nuthetal, Germany.

[13] German Center for Diabetes Research (DZD), 85764 München-Neuherberg, Germany.

## 1. Characterization of PC12 Z10

After ten hypoxia-reoxygenation cycles or parallel cultivation under normoxic conditions, PC12 Z10 and PC12 Z10 control cells were obtained and characterized with respect to morphological differences and changes in their growth pattern (Figure S1).

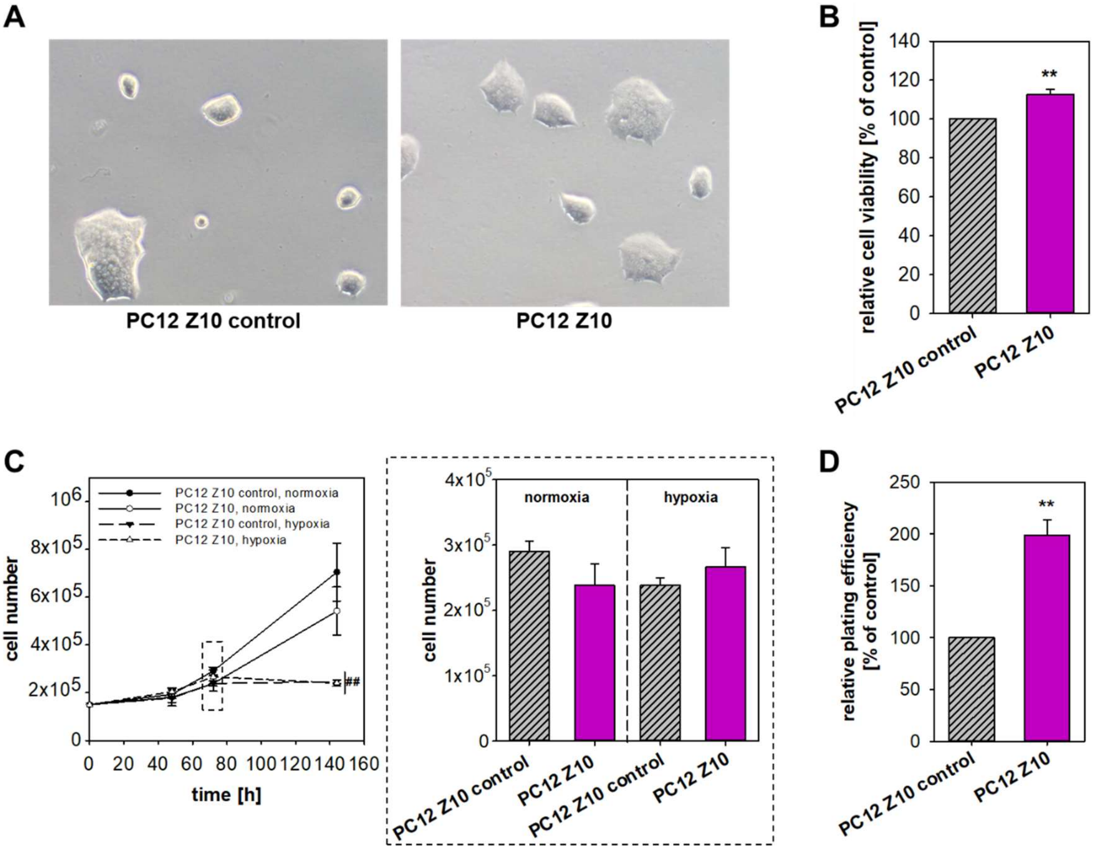


**Figure S1: Impact of ten recurrent cycles of hypoxia on the growth characteristics of PC12 cells**. (**A**) The established sub-cell line P12 Z10, obtained after ten recurrent cycles, showed no morphological changes compared to PC12 Z10 control cells analyzed by phase contrast microcopy. (**B**) PC12 Z10 cells revealed increased cell viability compared to the control sub-cell line. Twelve independent experiments (n=36). (**C**) The growth rate of PC12 Z10 cells was comparable to the PC12 Z10 control cells, but cultivation under hypoxic condition reduced the growth of both cells significantly. Six independent experiments (n=12). (**D**) Clonogenic survival assays revealed an enhanced plating efficiency of PC12 Z20 cells compared to the controls. Three independent experiments (n=3). Mean±SEM; Anova and Bonferroni post hoc test comparison vs. respective cells under normoxic condition ## p<0.001, vs. control cell line **p<0.001. Four to five independent experiments (n=16-24).

## 2. Treatment of hPheo1 cells with recurrent cycles of hypoxia

In addition to PC12 cells, hPheo1 cells were treated with recurrent cycles of hypoxia. The treatment scheme and morphological differences of the resulting cell lines are shown in Figure S1.

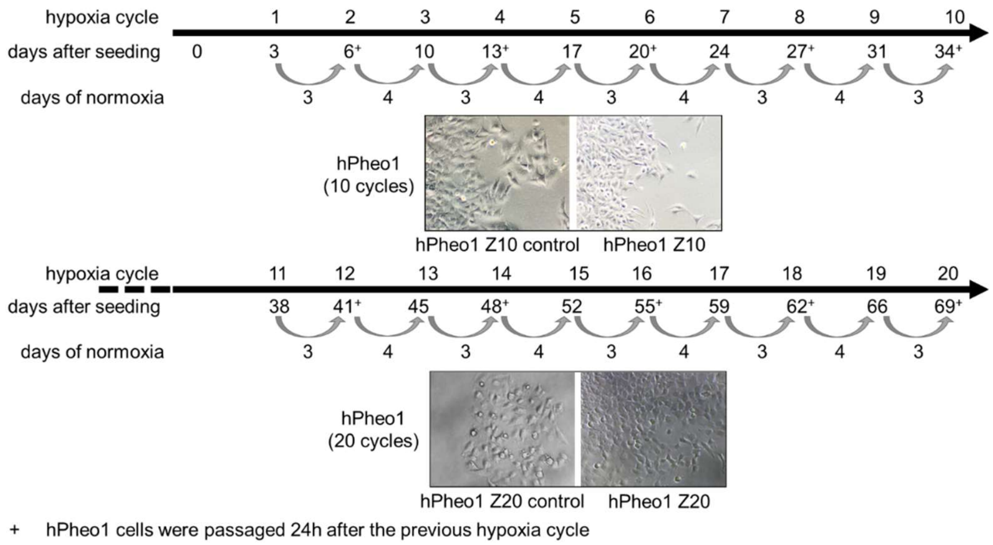


**Figure S2: Schematic representation of the experimental procedure and the time processes during generation of new hPheo1 sub-cell lines.** Cells were treated with recurring cycles of hypoxia (≤1% oxygen, 24 h hypoxia/cycle followed by a reoxygenation phase of three to four days). After 10 (hPheo1 Z10) and 20 (hPheo1 Z20) cycles, morphological changes in comparison to the control cells cultivated under normoxic conditions (hPheo1 Z10 control and hPheo1 Z20 control) were determined by phase contrast microcopy.

In contrast to PC12 cells, hPheo1 cells showed already some changes in their cellular behavior after 10 hypoxia cycles compared to the control cells cultivated under normoxic conditions (Figure S2). hPheo1 Z10 cells showed in trend an increased number of cells under normoxic condition, while this growth characteristic was not affected in the hPheo1 Z20 cells compared to the respective control cells (Figure S2A-B). The migration capacity of these cells remained comparable after 10 and 20 cycles of hypoxia (Figure S2C). The hPheo1 sub-cell line obtained after 10 cycles of hypoxia showed a significantly higher invasion capacity than the control cells, while differences in the hPheo1 Z20 were no longer significant (Figure S2D). hPheo1 Z10 cells attached significantly more to the extracellular matrix protein collagen compared with the control cell line (Figure S2E). This effect was also no longer significant in the cells generated after 20 hypoxia cycles. The adhesion ability to laminin was not altered in either cell line (Figure S2F). hPheo1 cells have a significantly higher growth rate than PC12 cells. These data suggest that phenotypic changes in response to recurrent treatment with hypoxia occur more rapidly in faster proliferating cells. Therefore, it is critical to study different numbers of cycles to establish comparable models.

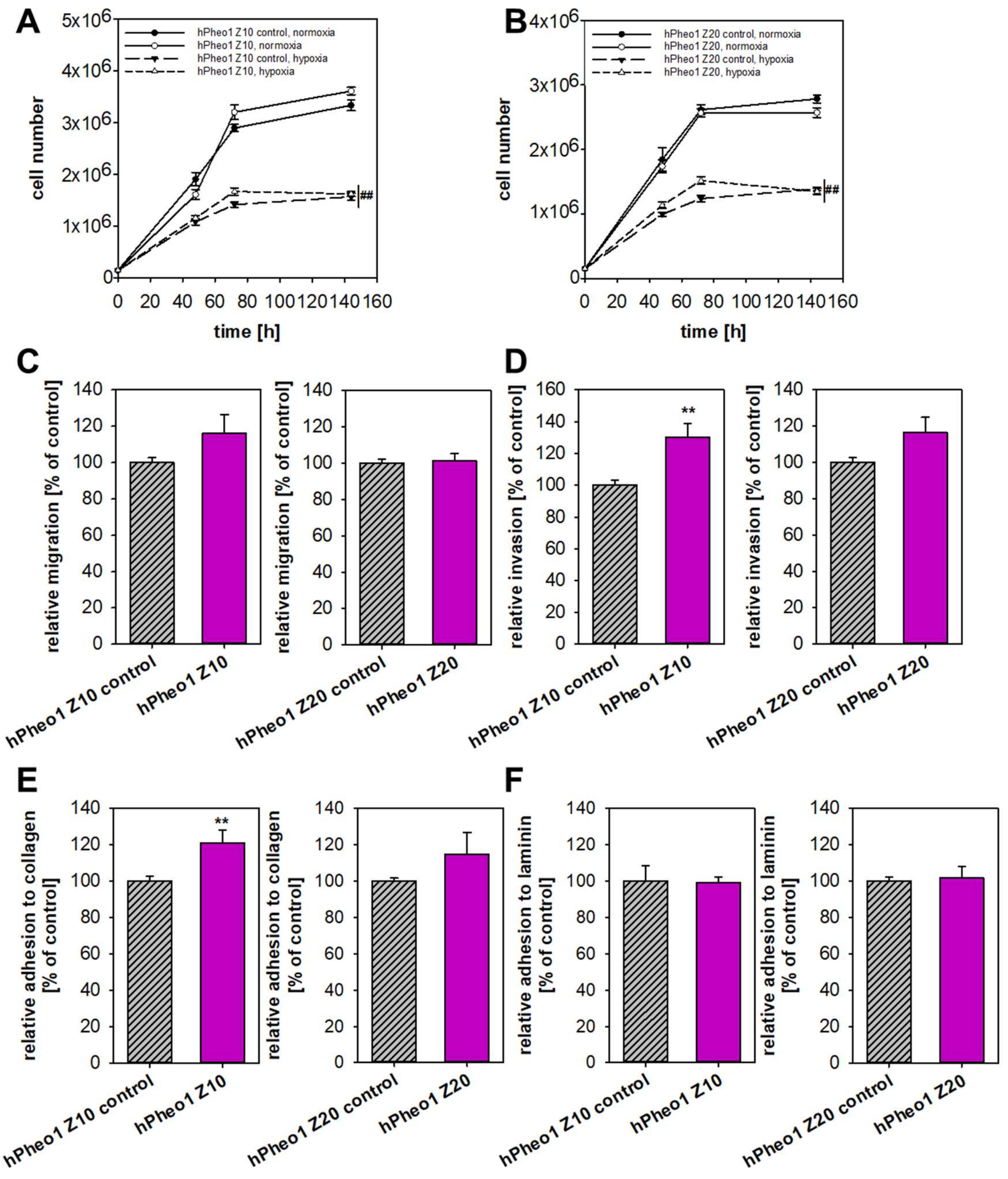


**Figure S3: Impact of recurrent cycles of hypoxia on the pro-metastatic behavior of hPheo1 cells**. (**A**) Ten (hPheo1 Z10) or (**B**) twenty (hPheo1 Z20) cycles of hypoxia showed no effect on the growth in comparison to the respective hPheo1 control cells under normoxic or hypoxic conditions. Mean±SEM; Anova and Bonferroni post hoc test comparison vs. respective cells under normoxic condition # p<0.05, ## p<0.001, vs. control cell line *p<0.05. Impact of recurrent cycles of hypoxia on the (**C**) migration and (**D**) invasion capacity as well as on adhesion ability to (**E**) collagen or (**F**) laminin of hPheo1 cells. Four to five independent experiments (n=16-24). Mean±SEM; unpaired t-test comparison vs. hPheo1 Z10 control or hPheo1 Z20 control * p<0.05, ** p<0.01.

## 3. Therapy response to common chemotherapeutics

### 3.1. Experimental details: Viability assay

To investigate the effect of different chemotherapeutics (cisplatin, doxorubicin, etoposide, paclitaxel, dacarbazine, cyclophosphamine, vincristine) on cell viability the CellTiter 96® Aqueous One Solution Cell Proliferation Assay (Promega, Mannheim, Germany) was used. In analogy to manufacturer's instructions cells were seeded in 96-well plates. After cell adhesion (24 h), cells were incubated for 24 h with different concentrations of the chemotherapeutics or DMSO as control. Afterwards cells were incubated with CellTiter 96® Aqueous One reagent (3.5 h) at 37°C and the absorption was measured at 492 nm by Spark® multimode microplate reader (Tecan Group Ltd., Männedorf, Switzerland). For the calculation of the half maximal effective concentration ($EC_{50}$) a dose-response curve by using the dose-response fit model of the SigmaPlot 12.5 software package (SYSTAT Software, San Jose, USA) was utilized.

### 3.2. Comparable responses to common chemotherapeutics of PC12 Z20 cells compared to control

Tumor hypoxia contributes to an overall diminished efficiency of chemotherapeutic agents [1]. We therefore investigated whether PC12 Z20 cells exhibit resistance to common chemotherapeutic agents compared to PC12 Z20 control cells (Table S1). We therefore treated the cells for 24 h with different concentrations of vincristine, etoposide, cisplatin, decarbazine, cyclophosphamine, paclitaxel or doxorubicine. Up to a concentration of 50 µM, none of the tested chemotherapeutic agents resulted in a 50% reduction of the viability (half maximal effective concentration; $EC_{50}$). Doxorubicin and etoposide had the strongest effect on the viability of both PC12 cell lines. We furthermore performed clonogenic survival assays, but no significant differences between both cell lines in response to vincristine, etoposide, cisplatin, dacarbazine, cyclophosphamine, paclitaxel, and doxorubicin were observed.

**Table S1: Impact of common chemotherapeutics on the viability of PC12 Z20 and PC12 Z20 control cells.**

| Compound | PC12 Z20 control $EC_{50}$ [µM] | PC12 Z20 control Viability 50 µM [%] | PC12 Z20 $EC_{50}$ [µM] | PC12 Z20 Viability 50 µM [%] |
|---|---|---|---|---|
| Vincristine | n.a. | 74.8 ± 3.2 | n.a. | 85.0 ± 2.8 |
| Etoposide | n.a. | 57.1 ± 3.1** | n.a. | 62.0 ± 3.3** |
| Cisplatin | n.a. | 109.9 ± 3.5 | n.a. | 110.6 ± 4.5 |
| Dacarbazine | n.a. | 91.6 ± 1.0 | n.a. | 91.3 ± 2.5 |
| Cyclophosphamine | n.a. | 96.1 ± 0.5 | n.a. | 105.5 ± 10.5 |
| Paclitaxel | n.a. | 94.3 ± 4.0 | n.a. | 86.2 ± 3.6 |
| Doxorubicin | n.a. | 66.6 ± 7.6 | n.a. | 51.1 ± 4.2*** |

$EC_{50}$: half maximal effective concentration; AVERAGE ± SEM, unpaired students t-test comparison vs. DMSO control, ** $p<0.005$, *** $p<0.001$. n.a., not achieved up to a concentration of 50 µM.

### 3.3. Comparable responses to common chemotherapeutics of hPheo1 Z10 cells compared to control

In addition, we analyzed the impact of vincristine, etoposide, cisplatin, decarbazine, cyclophosphamine, paclitaxel or doxorubicine on the viability of hPheo1 Z10 and hPheo1 Z10 control cells (Table S2). Similar to the PC12 Z20 cells, the strongest effect was measurable after treatment with etoposide, but no differences between hPheo1 Z10 ($EC_{50}$= 50.0 µM) and hPheo1 Z10 control ($EC_{50}$= 51.1 µM) cells were observed. Treatment with 50 µM vincristine also led to a significant reduction of the cell viability of both cell lines to a comparable extent. No effect on viability could be detected for the treatment with cisplatin, dacarbazine, cyclophosphamine, paclitaxel, or doxorubicin (Table S2). We furthermore investigated the impact of the chemotherapeutica on the new cell lines in clonogenic survival assays. After treatment with vincristine, etoposide, cisplatin, decarbazine, cyclophosphamine, paclitaxel or doxorubicine no differences between the relative plating efficiencies between both cell lines were observed (data not shown). With respect to the chemotherapeutic agents used, both PC12 Z20 and hPheo1 Z10 cells showed no resistance compared with control cells, but the overall response of the cells to the various treatments was generally lower than expected.

**Table S2: Impact of common chemotherapeutics on viability of hPheo1 Z10 and hPheo1 Z10 control cells.**

| | hPheo1 Z10 control | | hPheo1 Z10 | |
|---|---|---|---|---|
| **Compound** | **$EC_{50}$ [µM]** | **Viability 50 µM [%]** | **$EC_{50}$ [µM]** | **Viability 50 µM [%]** |
| Vincristine | n.a. | 61.8 ± 4.9 * | n.a. | 58.4 ± 4.4 * |
| Etoposide | 51.1 | 50.4 ± 13.3** | 50 | 47.8 ± 13.8** |
| Cisplatin | n.a. | 104.7 ± 6.8 | n.a. | 96.0 ± 5.5 |
| Dacarbazine | n.a. | 90.1 ± 3.5 | n.a. | 89.3 ± 2.4 |
| Cyclophosphamine | n.a. | 88.4 ± 1.9 | n.a. | 95.5 ± 5.1 |
| Paclitaxel | n.a. | 70.4 ± 4.6 | n.a. | 74.9 ± 6.0 |
| Doxorubicin | n.a. | 75.0 ± 12.8 | n.a. | 67.0 ± 14.1 |

EC50: half maximal effective concentration; AVERAGE ± SEM, unpaired students t-test comparison vs. DMSO control, * $p<0.05$, ** $p<0.001$. n.a., not achieved up to a concentration of 50 µM.

## 4. Therapy resistance of hPheo1 Z10 cell spheroids towards the potent targeted drug combination, everolimus and BYL719

We previously demonstrated that clinically relevant doses of the specific phosphatidylinositol-3-kinase α inhibitor BYL719 in combination with the mammalian target of rapamycin inhibitor everolimus showed synergistic effects on pheochromocytoma cell spheroids and primary cultures of human pheochromocytomas [2]. We therefore aimed to investigate the effect of this potent combination therapy on our new cell line models. Tumor cell spheroids are an ideal model for *in vitro* drug testing because they are characterized by an oxygen and nutrient gradient, further reflecting the tumor situation. Even after treatment with recurrent hypoxia cycles, PC12 cells do not form spheroids, which is why the following studies focus only on the newly established hPheo1 cell lines (only hPheo1 Z10 and hPheo1 Z10 control, because of the strongest phenotypic differences).

### 4.1 Experimental details: Spheroid assay

Generation and cultivation of hPheo1 Z10 and hPheo1 Z10 control cell spheroids was conducted as previously described for the hPheo1 wild type cells [3]. Four days after spheroid generation, spheroids were treated with BYL719 (5 µM) or sunitinib (2 µM) alone or in combination with everolimus (10 nM). Afterwards, spheroid growth was monitored [4].

### 4.2 hPheo1 Z10 cell spheroids showed resistance towards the combination therapy with everolimus and BYL719 compared with spheroids of the control cells

In line with the results in monolayer culture (Figure S2), hPheo1 Z10 and hPheo1 Z10 control show a comparable spheroid growth (Figure S3A). Treatment with everolimus (10 nM) or with the multitargeted receptor tyrosine kinase inhibitor sunitinib (2 µM) reduced the spheroid growth of both cell lines to a similar extent (Figure S3B). Simultaneous treatment with EVE and SUN led synergistic effects that were comparable between both cell lines (Figure S3C). Treatment with BYL719 (5 µM) diminished spheroid growth of both cell lines to a comparable extent (Figure S3D). hPheo1 Z10 cell spheroids showed a significant resistance towards the combination treatment with EVE and BYL compared to the control spheroids reflected in a significantly larger spheroid diameter (Figure S3E). The synergistic effect of the combined treatment persists indicating the superiority of this targeted combination therapy over single treatment.

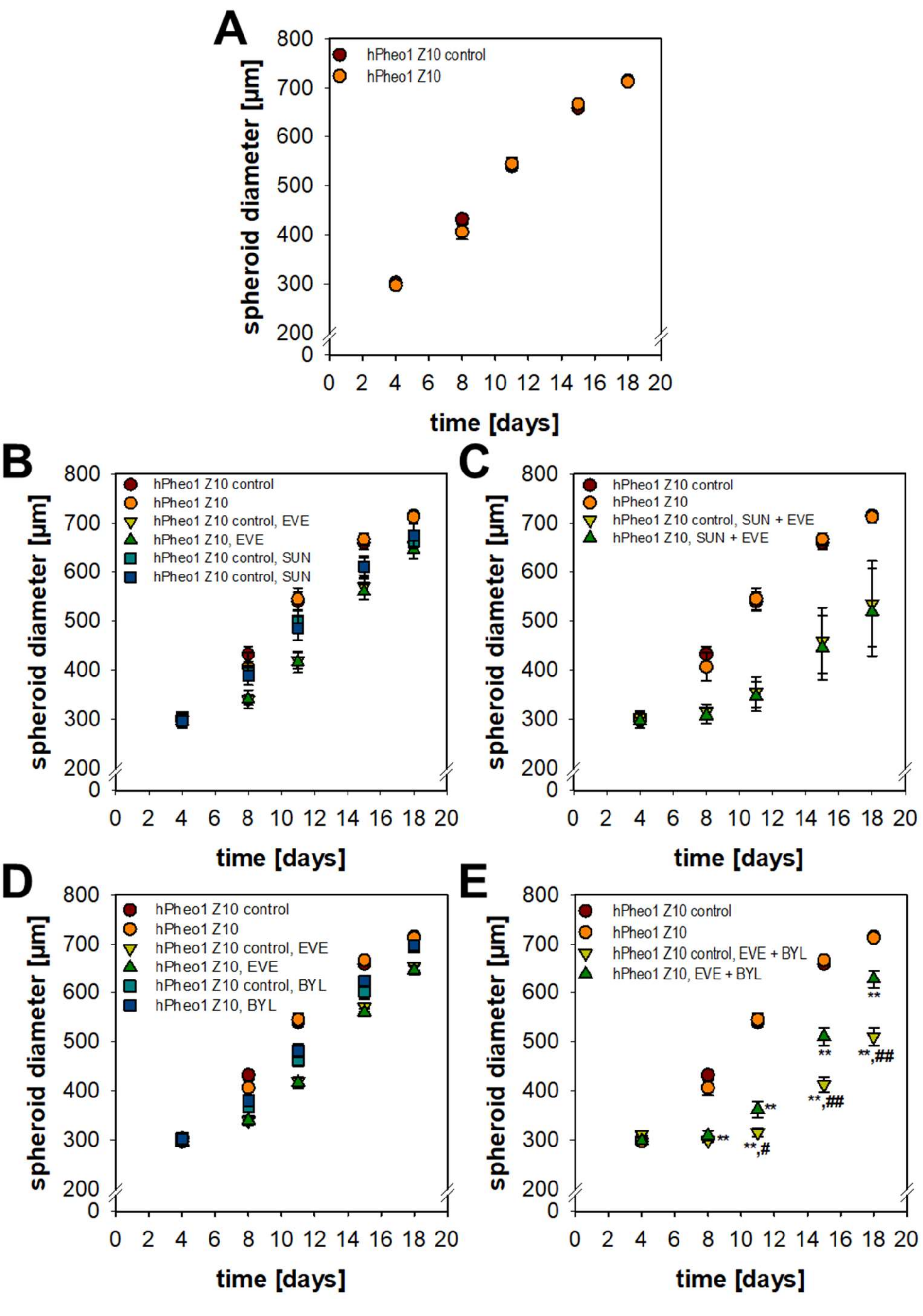


**Figure S4: Therapy response to targeted therapies of hPheo1 Z10 spheroids and their control cells.** (**A**) hPheo1 Z10 and hPheo1 Z10 control show a comparable spheroid growth. (**B**) Treatment with 10 nM everolimus (EVE) or 2 µM sunitinib (SUN) reduced the spheroid growth of both cell lines to a similar extent. (**C**) Simultaneous treatment with EVE and SUN led to comparable results in both cell lines. (**D**) Treatment with everolimus or 5 µM BYL719 (BYL) dimished the spheroid growth of both cell lines to a comparable extent. (**E**) hPheo1 Z10 cell spheroids showed a significant resistance towards the combination treatment with EVE and BYL compared to the control spheroids. Four to five different experiments (n=12-15). Mean±SEM. Anova and Bonferroni post hoc test comparison vs. hPheo1 Z10 control ** $p<0.001$; vs. treated hPheo Z10 control cells # $p<0.05$, ## $p<0.001$.

## 5. Gene expression analysis in PC12 Z20 cells

RNA-sequencing was performed to characterize expressional differences between PC12 Z20 cells and PC12 Z20 control cells. Table S3 summarizes the most up- or down regulated genes in PC12 Z20 cells.

**Table S3: Twenty most up- or down regulated genes identified via RNA sequencing in PC12 Z20 cells vs. PC12 control.**

| 20 most up-regulated genes | | | 20 most down-regulated genes | | |
|---|---|---|---|---|---|
| **Gene** | **Log2 fold change** | **p-value** | **Gene** | **Log2 fold change** | **p-value** |
| ***Vnn1*** | 4.29 | 1.37E-102 | ***Dcdc2*** | -2.82 | 2.05E-103 |
| ***Col5a2*** | 2.78 | 1.61E-96 | ***Etl4*** | -3.23 | 9.28E-84 |
| ***Pdgfrb*** | 1.59 | 2.04E-61 | ***Ltbp1*** | -2.40 | 5.17E-41 |
| ***Vim*** | 3.15 | 2.41E-57 | ***Atp8a1*** | -1.11 | 4.50E-23 |
| ***AABR07054614.1*** | 3.50 | 2.06E-53 | ***Slc16a2*** | -1.03 | 2.13E-19 |
| ***Igf1*** | 1.43 | 3.40E-42 | ***Igf2*** | -1.06 | 2.31-19 |
| ***Nhsl1*** | 1.68 | 2.43E-39 | ***Dhrs9*** | -1.21 | 2.03E-18 |
| ***St3gal1*** | 2.00 | 3.18E-39 | ***Cplane1*** | -0.71 | 1.72E-17 |
| ***Cgn*** | 2.34 | 5.05E-39 | ***Ica1*** | -0.67 | 9.73E-16 |
| ***Gata4*** | 5.99 | 1.61E-38 | ***Rassf9*** | -2.84 | 4.55E-15 |
| ***Vcan*** | 3.01 | 3.07E-37 | ***Wdr59*** | -0.64 | 3.94E-14 |
| ***Ptprz1*** | 2.69 | 8.89E-37 | ***Calhm5*** | -1.22 | 5.14E-13 |
| ***Tgfbr2*** | 6.19 | 9.31E-35 | ***Glcci1*** | -0.84 | 5.21E-13 |
| ***Drd2*** | 2.73 | 1.23E-33 | ***Ptger3*** | -0.63 | 7.74E-12 |
| ***Plin2*** | 1.68 | 4.18E-30 | ***Unc5c*** | -0.69 | 9.95E-12 |
| ***Tgfbi*** | 1.76 | 3.07E-29 | ***Npnt*** | -3.52 | 1.48E-11 |
| ***Ccn5*** | 2.41 | 1.80E-28 | ***Krt23*** | -2.44 | 1.70E-11 |
| ***Serpine2*** | 2.73 | 7.90E-28 | ***Sh2d4a*** | -4.45 | 4.40E-11 |
| ***Rapgef5*** | 1.66 | 1.85E-27 | ***Tex13b*** | -3.24 | 7.22E-11 |
| ***Col6a1*** | 2.16 | 5.09E-27 | ***Fxyd6*** | -0.57 | 3.71E-10 |

## 6. HIF2α expression in PC12 Z20 and PC12 Z20 control cells

SDS-PAGE and Western blot analysis were performed to investigate the differences between the new established sub-cell line and their corresponding control cell line. In addition, cobalt chloride ($CoCl_2$) was used to increase HIF2α stabilization in these cells. The results are shown in Figure S5 and experimental procedure is briefly described below.

After treatment of cells with 0.1 mM $CoCl_2$ or DMSO as control, cells were lysed and analyzed by SDS-PAGE and Western blot analysis as described previously [5]. After SDS-PAGE proteins were transferred to a nitrocellulose membrane. After blocking (5% skimmed milk powder plus 2% bovine serum albumin in TBS-T,

blocking solution), membranes were incubated with primary antibody anti-HIF2α (1:500; AF2997, R&D Systems, Inc.) and anti-Actin (1:1000; MAB1501R, Millipore, Massachusetts, USA) followed by incubation with the corresponding peroxidase-conjugated secondary antibody donkey anti-goat (1:5000, sc-2020, Santa Cruz Biotechnology) and goat anti-mouse (1:5000, sc-2005, Santa Cruz Biotechnology).

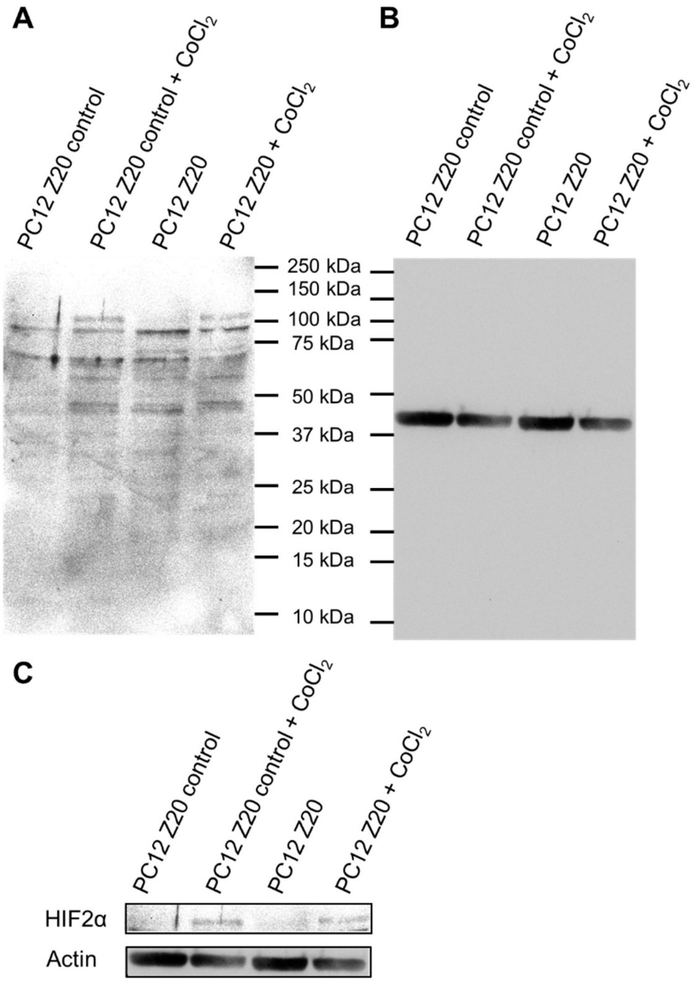


**Figure S5: HIF2α expression in PC12 Z20 and PC12 Z20 control cells.** HIF2α expression with or without $CoCl_2$ (0.1 mM, 24 h) treatment was analyzed by SDS-PAGE followed by a Western blot analysis. Immunological detection of (**A**) HIF2α and (**B**) Actin. (**C**) Representative section of the immunological detection of HIF2α and Actin. Three independent experiments.

## Reference


1. Shannon, A.M., et al., *Tumour hypoxia, chemotherapeutic resistance and hypoxia-related therapies.* Cancer treatment reviews, 2003. **29**(4): p. 297-307.
2. Fankhauser, M., et al., *Synergistic Highly Potent Targeted Drug Combinations in different Pheochromocytoma Models including Human Tumor Cultures.* Endocrinology, 2019.
3. Bechmann, N., et al., *HIF2α supports pro-metastatic behavior in pheochromocytomas/paragangliomas.* Endocrine-Related Cancer, 2020. **27**(11): p. 625-640.
4. Bechmann, N., et al., *Impact of extrinsic and intrinsic hypoxia on catecholamine biosynthesis in absence or presence of HIF2α in pheochromocytoma cells.* Cancers, 2019. **11**(5): p. 594.
5. Bechmann, N., et al., *Anti-Tumorigenic and Anti-Metastatic Activity of the Sponge-Derived Marine Drugs Aeroplysinin-1 and Isofistularin-3 against Pheochromocytoma In Vitro.* Marine drugs, 2018. **16**(5): p. 172.
